# Increased Immunogenicity of a Minimally Immunogenic Tumor after Cancer-Targeting Near Infrared Photoimmunotherapy

**DOI:** 10.3390/cancers12123747

**Published:** 2020-12-12

**Authors:** Hiroaki Wakiyama, Aki Furusawa, Ryuhei Okada, Fuyuki Inagaki, Takuya Kato, Yasuhiro Maruoka, Peter L. Choyke, Hisataka Kobayashi

**Affiliations:** Molecular Imaging Branch, Center for Cancer Research, National Cancer Institute, NIH, Bethesda, MD 20892, USA; Hiroaki.Wakiyama@nih.gov (H.W.); Aki.Furusawa@nih.gov (A.F.); Ryuhei.Okada@nih.gov (R.O.); Fuyuki.Inagaki@nih.gov (F.I.); Takuya.Kato@nih.gov (T.K.); ymaruoka@med.kyushu-u.ac.jp (Y.M.); pchoyke@mail.nih.gov (P.L.C.)

**Keywords:** near infrared photoimmunotherapy, non-immunogenic tumor, host anti-cancer immunity, immune checkpoint inhibitor, PD-1

## Abstract

**Simple Summary:**

Near-infrared photoimmunotherapy (NIR-PIT) is a highly selective cancer treatment that employs an antibody photoabsorber conjugate (APC) composed of a targeting monoclonal antibody (mAb) conjugated with a photoactivatable phthalocyanine-derivative dye. APC bound cancer cells are selectively destroyed upon NIR light exposure. Since NIR-PIT can induce selective immunogenic cell death, even in poorly immunogenic tumors, NIR-PIT has the potential to improve immunogenicity of tumor cells that could further enhance anti-tumor host immunity when combined with immune checkpoint inhibition. In this study, we showed that cancer-targeting NIR-PIT converted a minimally immunogenic tumor (MOC2-luc) into a highly immunogenic tumor by increasing the number and density of activated CD8+ T cells infiltrating into the tumor, leading to improved efficacy of the anti-PD-1 immune-checkpoint inhibitor.

**Abstract:**

Near-infrared photoimmunotherapy (NIR-PIT) is a highly selective cancer treatment that employs an antibody photoabsorber conjugate (APC) composed of a targeting monoclonal antibody (mAb) conjugated with a photoactivatable phthalocyanine-derivative dye. Once injected and allowed to bind to a tumor, the APC is activated by local near-infrared light which kills cancer cells and induces a strong immune response in the tumor microenvironment by unmasking of new tumor antigens emerging from damaged tumor cells. Due to its ability to incite an immune reaction, even in poorly immunogenic tumors, NIR-PIT has the potential to enhance immunogenicity in tumors especially after immune checkpoint inhibition. In this study, we employ a poorly immunogenic MOC2-luc syngeneic tumor model and evaluate the efficacy of cancer-targeting CD44-targeted NIR-PIT. Increased infiltration of CD8+ T cells observed after NIR-PIT suggested an enhanced immune environment. Next, we evaluated tumor progression and survival after the combination of CD44-targeted NIR-PIT and short-term administration of an anti-PD1 immune checkpoint inhibitor (ICI) to further activate CD8+ T cells. Additionally, in mice in which the tumors were eradicated by this combination therapy, a re-challenge with fresh MOC2-luc cells demonstrated failure of tumor implantation implying acquired long-term immunity against the cancer cells. Combination therapy decreased tumor progression and prolonged survival significantly. Therefore, we concluded that NIR-PIT was able to convert a minimally immunogenic tumor unresponsive to anti-PD-1 ICI into a highly immunogenic tumor responsive to anti-PD-1 ICI, and this therapy was capable of inducing long-term immunity against the treated cancer.

## 1. Introduction

Immune checkpoint inhibitors (ICIs) represent a rapidly expanding treatment class for cancer. However, it is often difficult to predict the response to treatment. The objective response rate is often suboptimal in patients with recurrent or metastatic cancers (11.2–31.7%), except for Hodgkin’s lymphoma (66.3–66.9%) [1]. This is probably because many tumors have an insufficient immune environment with poor T cell infiltration. In such cases, it is often difficult to recruit a large enough T cell population into the tumor after the therapy. Indeed, tumor-infiltrating CD8+ T cell density has been investigated as a biomarker of response to ICIs [2,3]. Thus, the relative absence of a pre-existing adaptive immune response poses a great challenge for ICI therapy in these tumors [4,5].

Near-infrared photoimmunotherapy (NIR-PIT) is a newly developed cancer treatment that uses an antibody-photoabsorber conjugate (APC) [6,7], the main components of which are a monoclonal antibody (mAb) against a surface antigen selectively expressed on cancer cells and a photoactivatable infrared phthalocyanine-derivative dye (IRDye700DX: IR700). Once injected into the patient’s body, the APC binds to the tumor cells over a period of time (typically within 24 h). When NIR light is irradiated to activate the dye, the photo-induced ligand release reaction of IR700 accompanied by morphological change of APC causes cell membrane damage and cancer cells are rapidly and selectively destroyed [8]. NIR-PIT induces a strong immune response in the tumor microenvironment (TME) due to the unmasking of new tumor antigens emerging from damaged tumor cells [9]. NIR-PIT has been shown to be effective in the clinical setting, with a first-in-human phase 1/2 clinical trial of NIR-PIT using cetuximab-IR700 (RM1929) targeting epidermal growth factor receptor (EGFR) in patients with inoperable head and neck squamous cell cancer successfully concluded in late 2017 [10]. Early results suggested that NIR-PIT is superior to existing second- and third-line therapies for recurrent head and neck cancers. A global phase 3 clinical trial of NIR-PIT using cetuximab-IR700 began in 2019 in patients with recurrent head and neck cancer who have failed at least two lines of therapy [11]. Much of the success of NIR-PIT is owed to the strong immune response it induces.

Since NIR-PIT can induce selective immunogenic cell death in cancer cells with minimal damage to immune cells in the TME [9], such immune cells are rapidly activated, reacting against released cancer antigens. Anti-tumor immunity includes adoptive immunity and innate immunity. Our previous study showed that the immune activation caused by NIR-PIT induced a multiclonal T cell response and increased tumor-infiltrating lymphocytes (TILs) [12]. Besides T-cell-mediated adoptive immunity, previous studies showed that NIR-PIT could have effects on innate immune system [13,14]. Therefore, we hypothesize that NIR-PIT could improve the response to ICI in a poorly immunogenic tumor.

In our previous studies, we reported the antitumor effects of the combination therapy of CD44-targeted NIR-PIT and PD-1 mAb in several syngeneic murine models [12]. However, poorly immunogenic tumors were not included in those studies. MOC2-luc is a CD44-expressing syngeneic murine oral cancer cell line that is known to be poorly immunogenic [15,16,17]. This study aims to investigate the possibility of CD44-targeted NIR-PIT in a poorly immunogenic tumor model, MOC2-luc, to induce additional antitumor immunity and sensitize it to anti-PD-1 ICI when given as a combination therapy.

## 2. Results

### 2.1. Immune Cell Infiltration in the Murine Syngeneic Tumor Models

**Figure 1:**
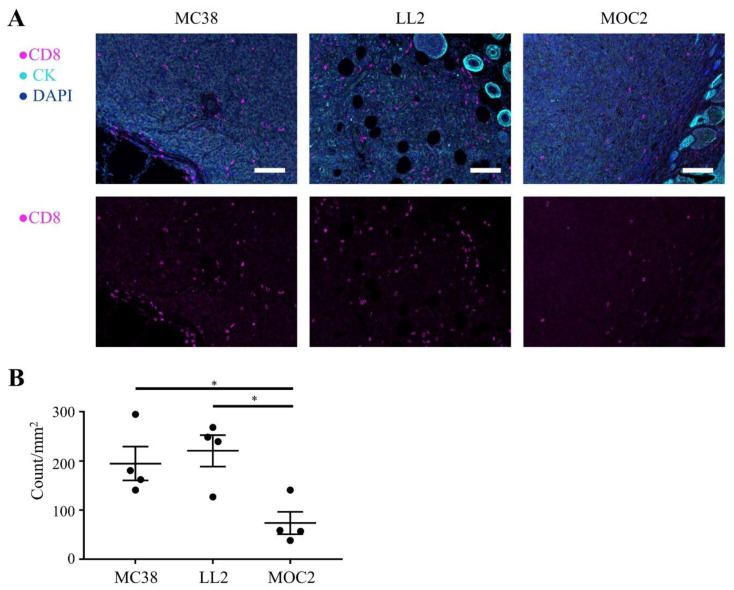
Tumor-infiltrating CD8+ T cells in luciferase-expressing tumors. Distribution of CD8+ T cells were assessed with multiplex immunohistochemistry (IHC). (**A**) Representative IHC images of luciferase-expressing MC38, LL2 and MOC2 tumors. Upper panels show composite images of CD8, pan-cytokeratin (CK) and DAPI staining, lower panels show single channel images of CD8 staining. CK was used to mark tumor tissue (×200, scale bar = 100 µm). The ring-like structures with high expression of CK are hair follicles. (**B**) CD8+ T cells within tumors were counted in multiplex IHC images. Data are shown as cell count per mm^2^ (*n* = 4; *, *p* < 0.05; one-way ANOVA followed by Tukey’s test).

We examined immune cell infiltration into tumor tissues by immunohistochemistry (IHC). First, we compared the number of CD8+ TILs in three murine syngeneic luciferase-expressing tumor models, MC38-luc, LL2-luc and MOC2-luc. The number of CD8+ TILs of MOC2-luc tumors was lower than the other two tumor models (*p* < 0.05; Figure 1A,B). Next, we compared the number of F4/80+ cells which include macrophages. Although statistically insignificant, the MOC2-luc tumor showed a tendency to have smaller number of F4/80+ cells within tumor tissue compared to the other two tumor models (Figure S1). These results suggested that the low immunogenicity of the MOC2-luc tumor was attributed to the low infiltration of immune cells which mediate both adoptive immunity and innate immunity. We also examined CD44 expression in tumor-infiltrating CD8+ T cells in a MOC2-luc tumor. We observed a mixture of CD44-positive and CD44-negative CD8+ T cells within MOC2-luc tumor tissue, suggesting that these CD44-positive CD8 T cells may be depleted upon CD44-targeted NIR-PIT (Figure S2).

### 2.2. Specific Binding of Anti-CD44-IR700 on MOC2-Luc Cells

**Figure 2:**
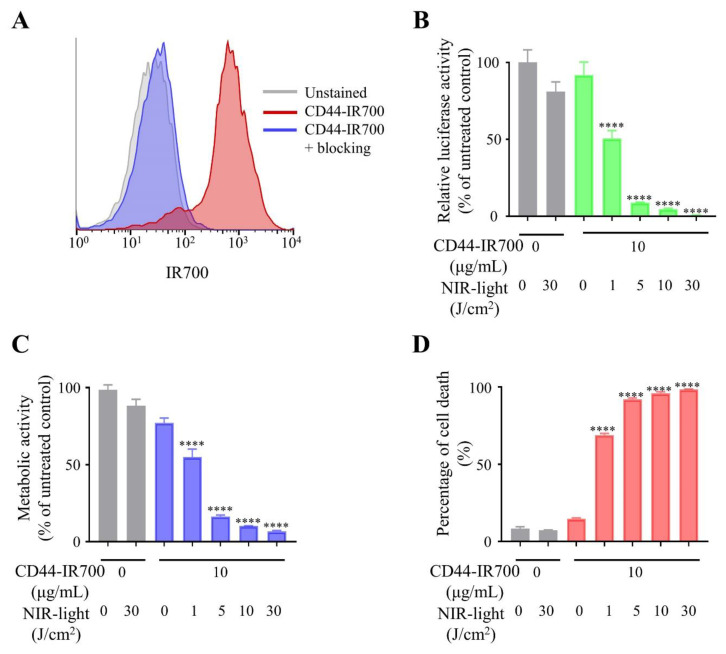
CD44-targeted NIR-PIT (near-infrared photoimmunotherapy) effectively killed MOC2-luc cells. (**A**) Binding of anti-CD44-IR700 to cell surface CD44 in MOC2-luc cells was examined with flow cytometry. CD44-blocking antibody was added to some wells to validate specific staining. Representative histograms are shown. (**B**) Relative luciferase activity in MOC2-luc cells was measured by bioluminescence imaging (*n* = 4; ****, *p* < 0.0001 vs. untreated control; one-way ANOVA followed by Tukey’s test). Each value represents means (% of control mean) ± SEM of independent experiments. (**C**) Metabolic activity measured by MTT assay (*n* = 4; ****, *p* < 0.0001 vs. untreated control; one-way ANOVA followed by Dunnett’s test). Each value represents means (% of control mean) ± SEM of independent experiments. (**D**) Membrane damage of MOC2-luc cells induced by NIR-PIT was measured using propidium iodide (PI) staining (*n* = 4; ****, *p* < 0.0001 vs. untreated control; one-way ANOVA followed by Tukey’s test). Each value represents means ± SEM of independent experiments.

After incubation with anti-CD44-IR700, IR700 fluorescence signal was detected on MOC2-luc cells by flow cytometry analysis (Figure 2A). This fluorescence signal was completely blocked by the addition of excess amount of unconjugated anti-CD44 mAb. These results indicated that anti-CD44-IR700 specifically binds to CD44 expressed on the cell surface of MOC2-luc cells.

### 2.3. In Vitro Effect of CD44-Targeted NIR-PIT against MOC2-Luc Cells

The cytotoxic effects of CD44-targeted NIR-PIT on MOC2-luc cells were quantitatively assessed by three types of cell viability assays. After the CD44-targeted NIR-PIT on MOC2-luc cells, bioluminescence imaging (BLI) demonstrated decreased luciferase activity in a light dose-dependent manner (Figure 2B). 3-(4,5-Dimethyl-2-thiazolyl)-2,5-diphenyl-2H-tetrazolium bromide (MTT) assay showed reduction of the proportion of live cells in a light dose-dependent manner (Figure 2C). PI (propidium iodide) staining showed increase of cell membrane damage in a light dose-dependent manner (Figure 2D). In all three assays, live cells were nearly undetectable after CD44-targeted NIR-PIT with 30 J/cm^2^ NIR light, whereas NIR light irradiation at 30 J/cm^2^ without anti-CD44-IR700 did not show significant differences in cell viability (Figure 2B–D). These results demonstrated that CD44-targeted NIR-PIT can efficiently kill the MOC2-luc cells.

### 2.4. In Vivo Effect of CD44-Targeted NIR-PIT against MOC2-Luc Tumor

**Figure 3:**
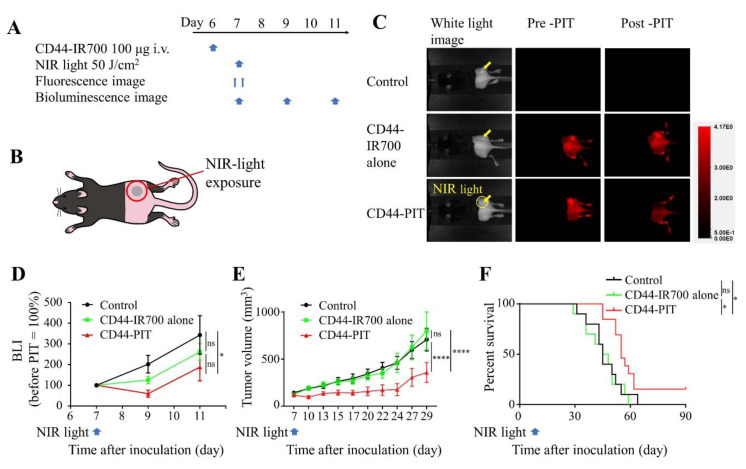
CD44-targeted NIR-PIT treated MOC2-luc tumors with limited success. CD44-targeted NIR-PIT was tested on MOC2-luc tumor-bearing mice. Control, no treatment; CD44-IR700 alone, i.v. injection of anti-CD44-IR700 only; CD44-PIT, i.v. injection of anti-CD44-IR700 with NIR light exposure. (**A**) NIR-PIT regimen. Bioluminescence and fluorescence images were obtained at each time point as indicated. (**B**) Laser-light exposure. NIR light was administered on the tumor only. (**C**) In vivo IR700 fluorescence imaging of tumor-bearing mice before and after the NIR-PIT. The yellow arrows indicate the tumor locations. (**D**) In vivo BLI of tumor-bearing mice before and after NIR-PIT. Luciferase activity was quantified and shown in relative percentage to the signal intensity before treatment in three treatment groups (*n* = 10–13; *, *p* < 0.05; one-way ANOVA followed by Tukey’s test). (**E**) Tumor growth curves (*n* = 10–13; ****, *p* < 0.0001; one-way ANOVA followed by Tukey’s test). (**F**) Kaplan–Meier survival analysis following NIR-PIT treatment (*n* = 10–13; *, *p* < 0.05; log-rank test followed by Bonferroni correction). Each value in D and E represents means ± SEM of independent experiments.

The treatment efficacy of CD44-targeted NIR-PIT against MOC2-luc tumors was examined in vivo in a subcutaneously allografted mice model. The treatment schedule and imaging regimen are shown in Figure 3A,B. The fluorescent imaging showed an accumulation of fluorescent signal in the MOC2-luc tumor indicating that anti-CD44-IR700 was successfully delivered and bound to MOC2-luc tumor (Figure 3C). After irradiation of 50 J/cm^2^ of NIR light, the IR700 fluorescence signal in MOC2-luc tumors almost disappeared, indicating that this therapeutic light dose was sufficient to induce complete structural change of IR700 dye attached to anti-CD44 antibody [8]. The cell activity of MOC2-luc cells was monitored with BLI. The BLI signal of CD44-targeted NIR-PIT group decreased after the treatment and was significantly lower compared to that of the control group 4 days after the treatment (day 11) (*p* < 0.05; Figure 3D). Tumor growth was significantly inhibited in the CD44-targeted NIR-PIT group compared with all other groups at day 29 (*p* < 0.0001; Figure 3E). Significantly prolonged survival was achieved in the CD44-targeted NIR-PIT group (*p* < 0.05; Figure 3F). No significant therapeutic effect was observed in the anti-CD44-IR700 alone group. A total of 2 out of 13 mice showed complete remission of tumors. These results demonstrate that CD44-targeted NIR-PIT with a single exposure of NIR light is effective against MOC2-luc tumors. However, complete remission of tumors after CD44-targeted NIR-PIT was uncommon.

### 2.5. CD8+ TILs and Innate Immune Cells after CD44-Targeted NIR-PIT in MOC2-Luc Tumor

**Figure 4:**
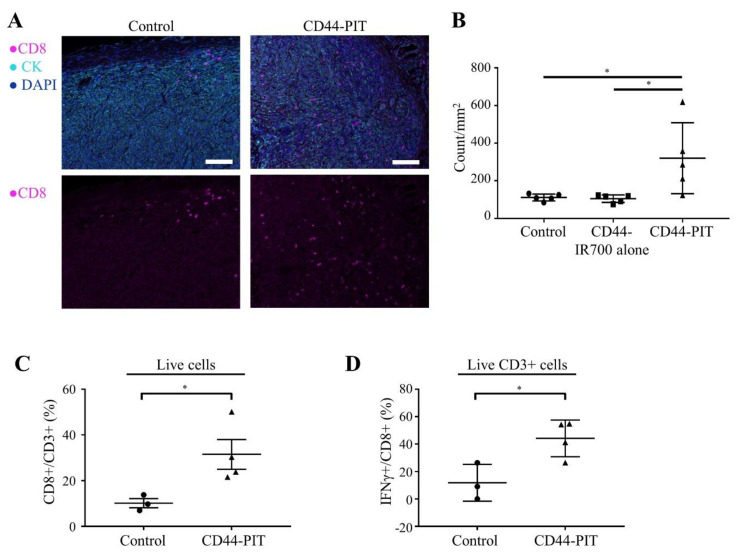
CD44-targeted NIR-PIT increased tumor infiltration of activated CD8+ T cells. (**A**,**B**) Distribution of CD8+ T cells was assessed with multiplex immunohistochemistry (IHC). (**A**) Representative images of multiplex IHC. Upper panels show composite images of CD8, pan-cytokeratin (CK) and DAPI staining, lower panels show single channel images of CD8 staining. CK was used to mark tumor tissue. Tumors were extracted 4 days after NIR-PIT. (×200, scale bar = 100 µm). (**B**) Cell number of CD8+ T cells within tumor tissue was counted in multiplex IHC images. Data are shown as cell count per mm^2^ (*n* = 5; *, *p* < 0.05; one-way ANOVA followed by Tukey’s test). (**C**) CD8+/CD3+ ratio in tumor microenvironment was examined with flow cytometry. Tumors were extracted 5 days after NIR-PIT (*n* = 3–4; *, *p* < 0.05; unpaired t-test). (**D**) IFN-γ+/CD8+ ratio of tumor microenvironment was examined with flow cytometry. Tumors were extracted 5 days after NIR-PIT (*n* = 3–4; *, *p* < 0.05; unpaired t-test). Control, no treatment; CD44-IR700 alone, i.v. injection of anti-CD44-IR700 only; CD44-PIT, i.v. injection of anti-CD44-IR700 with NIR light exposure. Each value represents single experiment, shown with means ± SEM.

Since CD44-targeted NIR-PIT showed an effective therapeutic response in a MOC2-luc tumor, we analyzed the tumor infiltration of CD8+ T cells before and after CD44-targeted NIR-PIT. IHC showed increased CD8+ TILs after CD44-targeted NIR-PIT (Figure 4A,B). Flow cytometry also showed the increase in CD8+/CD3+ ratio in the treated tumor bed (Figure 4C). Increase of IFN-γ+/CD8+ ratio after CD44-targeted NIR-PIT showed augmented CD8+ T cell activation (Figure 4D). These results indicated that CD44-targeted NIR-PIT improved the immunologically “cold” MOC2-luc tumor microenvironment. This made us hypothesize that the therapeutic effect of the anti-PD-1 ICI could be enhanced when combined with CD44-targeted NIR-PIT. On the other hand, there was no significant change between control group and CD44-PIT group in the number of F4/80+, Ly6G+CD11b+ or Gzmb+CD3− cells which include macrophages, granulocytes and NK cells, respectively (Figure S3).

### 2.6. In Vivo Effect of Combined CD44-Targeted NIR-PIT and Anti-PD-1 mAb against MOC2-Luc Tumor

**Figure 5:**
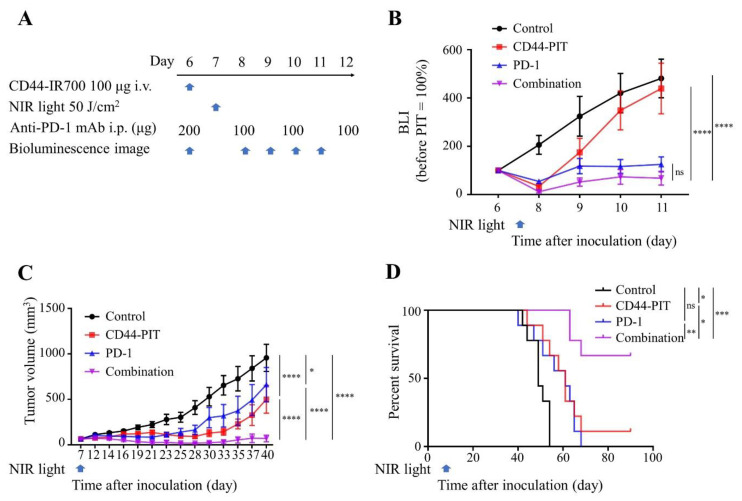
CD44-targeted NIR-PIT combined with short term anti-PD-1 ICI yielded CR (complete remission) to MOC2-luc tumors. Combination of CD44-targeted NIR-PIT and anti-PD-1 ICI was tested on MOC2-luc tumor-bearing mice. Control, no treatment; CD44-PIT, i.v. injection of anti-CD44-IR700 with NIR light exposure; PD-1, i.p. injection of anti-PD-1 only; combination, i.v. injection of anti-CD44-IR700 with NIR light exposure and i.p. injection of anti-PD-1. (**A**) NIR-PIT regimen. Each treatment timing and doses are indicated. Bioluminescence images were obtained at each time point indicated. (**B**) The luciferase activity was quantified and shown in relative percentage to the signal intensity before treatment in four groups (*n* = 9; ****, *p* < 0.0001; one-way ANOVA followed by Tukey’s test). (**C**) Tumor growth curves (*n* = 9; *, *p* < 0.05; ****, *p* < 0.0001; one-way ANOVA followed by Tukey’s test). (**D**) Kaplan–Meier survival analysis following NIR-PIT treatment (*n* = 9; *, *p* < 0.05, **, *p* < 0.01, ***, *p* < 0.001; log-rank test followed by Bonferroni correction). Each value in B and C represents means ± SEM of independent experiments.

We next tested if the improved immune environment caused by CD44-targeted NIR-PIT can sensitize MOC2-luc tumor to the ICI. The treatment efficacy of CD44-targeted NIR-PIT combined with anti-PD-1 mAb therapy was examined in vivo in a subcutaneously allografted MOC2-luc tumor model. Figure 5A shows the regimen and imaging schedule. The drop in BLI signal was greatest for the combination therapy group compared to that of the control or CD44-targeted NIR-PIT only group at day 11 (*p* < 0.0001; Figure 5B). Post-treatment tumor volume of the combination group was significantly lower than that of other 3 groups at day 40 (*p* < 0.0001; Figure 5C). Moreover, combination therapy significantly suppressed tumor growth and yielded superior rates (6 of 9 or 66.7%) of complete remission compared to either therapy alone. This resulted in significantly prolonged survival compared with the other 3 groups (vs Control group *p* < 0.001, vs CD44-targeted NIR-PIT group *p* < 0.05, vs. PD-1 group *p* < 0.01; Figure 5D). Although short term tumor growth inhibition was seen following anti-PD-1 ICI monotherapy (Figure 5B,C), the anti-PD-1 ICI monotherapy group showed no significant prolongation in overall survival compared with the control group (Figure 5D).

### 2.7. Resistance to Re-Challenge with MOC2-Luc Cells Following Complete Remission with Combination CD44-Targeted NIR-PIT and Anti-PD-1 mAb Treatment

**Figure 6:**
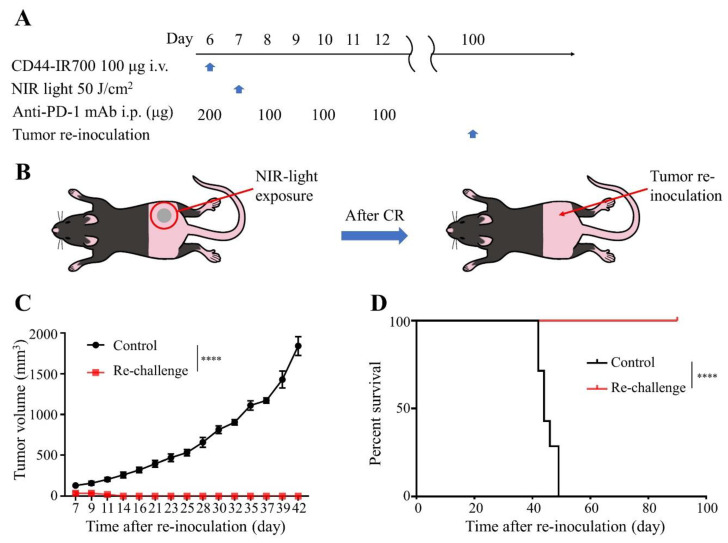
CD44-targeted NIR-PIT combined with short term anti-PD-1 ICI yielded long-term immunity against MOC2-luc tumors. The mice that achieved complete remission (CR) with the combination therapy were re-inoculated with MOC2-luc tumor 100 days after the first inoculation. (**A**) The regimen of tumor re-challenge. (**B**) Diagram of mice receiving re-inoculation of MOC2-luc cells. (**C**) Tumor growth curves (*n* = 7; ****, *p* < 0.0001; one-way ANOVA followed by Tukey’s test). (**D**) Kaplan–Meier survival analysis (*n* = 7; ****, *p* < 0.0001; log-rank test). Control, newly injected MOC2-luc tumor; Re-challenge, re-injected MOC2-luc tumor after CR with the combination therapy. Each value in C represents means ± SEM of independent experiments.

To assess acquired immunologic memory after combination therapy with CD44-targeted NIR-PIT and anti-PD-1 ICI, we re-challenged the mice that had a complete remission with re-inoculation of MOC2-luc cells (Figure 6A,B). Inoculated MOC2-luc tumors grew in all the control mice; in contrast, in the re-challenged mice, no tumor was established (Figure 6C, survival in Figure 6D). This result demonstrated that immunological memory against MOC2-luc cells was successfully established after the combination therapy.

## 3. Discussion

In this study, we showed that CD44-targeted NIR-PIT suppressed tumor growth and increased activation and tumor infiltration of CD8+ T cells resulting in a complete remission in approximately 15% of MOC2-luc tumor-bearing mice. As expected from “cold” immune environment of MOC2-luc tumor, anti-PD-1 ICI alone was not effective enough to achieve complete remission in any MOC2-luc tumor-bearing mice. However, a combination of CD44-targeted NIR-PIT and anti-PD-1 mAb was more effective compared to monotherapies with greater reduction of tumor progression, prolonged survival and an increased complete remission rate as high as 67%. Moreover, this combination therapy has shown to produce immunological memory against MOC2-luc tumors. This was considered to be because NIR-PIT converted a poorly immunogenic tumor into an immunogenic tumor, making it more susceptible to ICI. Since clinical outcomes after ICI therapy are often suboptimal in many cancers [1,18], NIR-PIT may be a way to transform “non-inflamed” or “cold” tumors into immunologically “hot” tumors [4,5,19].

Previous work has also shown that CD44-targeted NIR-PIT combined with anti-PD-1 mAb therapy can provide good outcomes for some syngeneic mouse tumors [12]. However, these tumors are known to be immunogenic, displaying high levels of lymphocyte infiltration. MOC2 cells are well known to be minimally immunogenic and MOC2 tumors show minimal lymphocyte infiltration. It is not surprising that they do not respond to ICIs [16]. MOC2-luc cells are engineered to express luciferase, which could potentially act as a tumor-specific antigen (TSA) and thus could contribute to the immunogenicity of NIR-PIT. However, this study showed that MOC2-luc tumor also showed minimal lymphocyte infiltration among luciferase-expressing tumor models and anti-PD-1 ICI produced no complete remission, which suggested that even with the added antigen of luciferase, MOC2-luc tumors are still poorly immunogenic [16,17].

This study and our other works have demonstrated the on-target cytotoxic effects of NIR-PIT which induce immunogenic cell death (ICD), thus inducing dendritic cell maturation [9,12]. ICD associated with NIR-PIT depends heavily on the presence of tumor target antigens (TTAs), some of which have been previously identified but many of which are unknown [9]. CD44 is overexpressed in various cancers; however, it is also expressed on non-cancer cells in the TME, including some populations of activated lymphocytes [20,21]. Our previous study showed that CD44-targeted NIR-PIT indeed killed CD44-expressing CD8+ T cells, sparing CD44-negative CD8+ T cells which can be activated later on [22]. We actually observed additional multiclonal T cell expansion and infiltration after CD44-targeted NIR-PIT [12]. In this study, only a part of CD8+ TILs were CD44 positive, therefore the effect of CD44-targeted NIR-PIT against CD8+ TILs would be limited. We also specifically showed that CD44-targeted NIR-PIT increased intra-tumoral CD8+ T cells and IFN-γ+ CD8+ T cells. This allowed the combination therapy of CD44-targeted NIR-PIT and anti-PD-1 ICI to be more effective than anti-PD-1 mAb ICI alone in MOC2-luc tumors. Thus, despite the naturally poor immunogenicity of MOC2-luc cells, CD44-targeted NIR-PIT could elicit host anti-cancer immunity in these “cold” tumors. 

Considering that the number of tumor-infiltrating F4/80+ cells was lower in MOC2-luc tumors than MC38-luc or LL2-luc tumors, the poorly immunogenic nature of the MOC2-luc tumor might be attributable to an insufficient innate immune system as well as T-cell-mediated adoptive immunity. Previous studies showed that NIR-PIT could affect the innate immune system [13,14]. However, in this study the number of innate immune cells did not change significantly after CD44-targeted NIR-PIT for MOC2-luc tumor, thus we concluded that T-cell-mediated adoptive immunity mainly contributed to the anti-tumor immunity.

One additional mechanism by which combination therapy likely improves ICI treatment efficacy is through an NIR-PIT-associated phenomenon known as the super enhanced permeability and retention (SUPR) effect [23]. Because APCs initially localize to perivascular tumor tissue due to their relatively large size, NIR-PIT tends to preferentially kill tumor cells adjacent to vessels, leading to a dramatic, if short-lived, increase in vascular permeability as the perivascular space enlarges and tumor vessels dilate. This effect differs from the enhanced permeability and retention (EPR) effect commonly observed in untreated tumor vasculature and is much more striking in magnitude. Thus, the SUPR effect could facilitate delivery of anti-PD-1 mAb within the tumor that can partly explain synergistic effects of these two treatments. 

In vitro, CD44-targeted NIR-PIT was highly effective in cell killing of MOC2-luc cells and there was no cytotoxicity with NIR laser light exposure alone. These results are consistent with previous work that used a light-emitting diode (LED) light source [17]. On the other hand, we previously reported on the antitumor effects of CD44-targeted NIR-PIT in syngeneic murine models of oral cancer using two doses of LED light (50 J/cm^2^ and 100 J/cm^2^), which resulted in no complete remission [17]. In this study, we achieved complete remission in 2 of 13 mice using a similar regimen of NIR-PIT. This difference could be attributed to the difference in the light source. In this study we used a laser light source instead of LEDs. Because laser light is more tightly monochromatic than LED light, a single exposure of 50 J/cm^2^ of NIR laser light is sufficient to deliver enough photon flux at the excitation wavelength of 690nm. These results support previous work that suggests that NIR-PIT using a laser system is approximately 3-fold more effective than that with an LED at the same energy dose [24].

## 4. Materials and Methods

### 4.1. Reagents

The water-soluble, silica-phthalocyanine derivative IRDye 700DX NHS ester (IR700) was obtained from LI-COR Biosciences (Lincoln, NE, USA). An anti-mouse/human CD44 mAb (clone IM7) and an anti-mouse PD-1 (CD279) mAb (clone RMP1-14) were purchased from Bio X Cell (West Lebanon, NH, USA). All other chemicals were of reagent grade.

### 4.2. Synthesis of IR700-Conjugated Anti-CD44 mAb

Conjugation of dye with mAb was performed according to a previous report [25]. In brief, anti-CD44 mAb (1.0 mg, 6.7 nmol) was incubated with IR700 NHS ester (66.8 μg, 34.2 nmol) in 0.1 mol/L Na_2_HPO_4_ (pH 8.6) at room temperature for 1 h. The mixture was purified with a Sephadex G25 column (PD-10; GE Healthcare, Piscataway, NJ, USA). The protein concentration was determined with a Coomassie Plus Protein Assay Kit (Thermo Fisher Scientific, Waltham, MA, USA) by measuring the absorption at 595 nm with UV-Vis (8453 Value System; Agilent Technologies, Santa Clara, CA, USA). The concentration of IR700 was measured by absorption at 689 nm with spectroscopy to confirm the number of fluorophore molecules conjugated to each mAb. The synthesis was controlled so that an average of two IR700 molecules was bound to a single antibody as previously described [17]. We abbreviate IR700-conjugated anti-CD44 mAb as anti-CD44-IR700.

### 4.3. Cell Culture

MC38 cells (murine colon cancer) were generously provided by Dr. Thomas Waldmann, NIH. LL/2 cells (murine Lewis lung carcinoma) were purchased from ATCC (Rockville, MD, USA) and MOC2 cells (murine oral carcinoma) were purchased from Kerafast (Boston, MA, USA). All three cells stably expressed luciferase via stable transduction with RediFect Red-Fluc lentivirus from PerkinElmer (Waltham, MA, USA) per manufacturer recommendations. Luciferase-expressing MC38, LL2 and MOC2 cancer cell lines, abbreviated as MC38-luc, LL2-luc and MOC2-luc, respectively, were used in this study. MC38-luc and LL2-luc cells were cultured in RPMI 1640 medium (Thermo Fisher Scientific, Waltham, MA, USA) supplemented with 10% fetal bovine serum, 100 IU/mL penicillin and 100 μg/mL streptomycin (Thermo Fisher Scientific, Waltham, MA, USA). MOC2-luc cells were cultured in media as previously described [17]. Cells were maintained in culture for no more than 30 passages and tested for mycoplasma.

### 4.4. Animal Model

All in vivo procedures were approved by the local Animal Care and Use Committee (NCI MIP-003). Six- to eight-week-old female wild-type C57BL/6 mice (strain #000664) were purchased from The Jackson Laboratory (Bar Harbor, ME, USA). Tumors were established via subcutaneous injection of 1 × 10^6^ cells in the caudal flank. For NIR-PIT treatments and fluorescence/bioluminescence imaging (BLI), mice were anesthetized with inhaled 2% to 3% isoflurane and/or via intraperitoneal injection of 0.75 mg of sodium pentobarbital (Nembutal Sodium Solution, Ovation Pharmaceuticals Inc., Deerfield, IL, USA). The hair overlying the tumor site was removed prior to NIR laser-light irradiation and imaging studies. For determination of tumor volume, the greatest longitudinal diameter (length) and the greatest transverse diameter (width) were measured with a caliper. Tumor volume was calculated as follows: tumor volume = length × width^2^ × 0.5. Tumor size was measured three times a week until the tumor volume reached 2000 mm^3^ or the length reached 2 cm, whereupon the mice were euthanized with inhalation of carbon dioxide gas.

### 4.5. Cell-mAb Binding Analysis

The specific binding of anti-CD44-IR700 to MOC2-luc cells was evaluated by flow cytometry. MOC2-luc cells (2 × 10^5^) were seeded into 12-well plates and incubated for 24 h. Medium was replaced with fresh culture medium containing 10 μg/mL of anti-CD44-IR700 and incubated for 1 h at 37 °C. After washing with phosphate buffered saline (PBS), fluorescence of IR-700 was analyzed with flow cytometer (FACSCalibur, BD Biosciences, San Jose, CA, USA) and CellQuest software (BD Biosciences, San Jose, CA, USA). To confirm the specific binding of anti-CD44-IR700, 10 times the amount of nonconjugated anti-CD44 mAb was added to a part of the samples (=blocking) 1 h prior to the administration of the anti-CD44-IR700.

### 4.6. In Vitro NIR-PIT

MOC2-luc cells were seeded at 2 × 10^5^ per well in quadruplicate onto 12-well plates in 2 mL of medium and incubated for 24 h. Cells were incubated with fresh culture medium containing 10 μg/mL of anti-CD44-IR700 for 1 h at 37 °C. After washing with PBS, phenol-red-free medium was added. NIR laser-light (690 nm, 150 mW/cm^2^) using an ML7710 laser system (Modulight, Tampere, Finland) was applied at 0, 1, 5, 10 and 30 J/cm^2^. One hour after NIR-PIT, the cytotoxic effects of NIR-PIT with anti-CD44-IR700 were determined by three types of cell viability assays as follows: For bioluminescence imaging (BLI), 500 μL of 150 μg/mL D-luciferin-containing media (Gold Biotechnology, St. Louis, MO, USA) was administered to PBS-washed cells and images were obtained on a BLI system (Photon Imager; Biospace Lab, Nesles la Vallée, France). Regions of interest (ROIs) were placed on each entire well and the luciferase activity (photons/min) was calculated using M3 Vision Software (Biospace Lab, Nesles la Vallée, France) [26]. For relative quantification, the value of luciferase activity in each group was normalized to the untreated control. For the MTT assay, medium was removed and 500 μL of MTT reagent (SIGMA Aldrich, St. Louis, MO, USA; 0.5 mg/mL) was added to each well. After 1 h of incubation at 37 °C, the supernatant was removed and 500 μL of 2-propanol was added to each well to dissolve the crystal formazan dye. After transferring 100 μL of the supernatant to each 96-well plate, absorbance was measured at 570 nm on a microplate reader (Synergy H1; BioTek, Winooski, VT, USA). For relative quantification, the value of absorbance in each group was normalized to the untreated control. For PI flow cytometric assay, cells were harvested with a cell scraper and stained with 1 μg/mL propidium iodide (PI) (Life Technologies, Carlsbad, CA, USA). The percentage of PI-stained cells was analyzed by BD FACSCalibur (BD Biosciences, San Jose, CA, USA) using FlowJo software (FlowJo LLC, Ashland, OR, USA).

### 4.7. In Vivo NIR-PIT

To evaluate the efficacy of CD44-targeted NIR-PIT, tumor-bearing mice were randomized into 3 groups as follows: (i) no treatment (Control); (ii) 100 μg of anti-CD44-IR700 i.v., no NIR laser-light exposure (CD44-IR700 alone); (iii) 100 μg of anti-CD44-IR700 i.v., with NIR laser-light (CD44-PIT). Anti-CD44-IR700 was intravenously (i.v.) administered 6 days after tumor inoculation and NIR laser-light (690 nm, 150 mW/cm^2^, 50 J/cm^2^) was administered the following day. Dorsal fluorescence images of IR700 were obtained with the 700 nm fluorescence channel of the Pearl Imager (LI-COR Biosciences, Lincoln, NE, USA). The images were taken pre- and post-NIR-PIT. Pearl Cam Software (LI-COR Biosciences, Lincoln, NE, USA) was used for analyzing fluorescence. Regions of interest (ROIs) were placed on the tumor. Acute effects of the treatments were evaluated with BLI. For BLI, 200 μL of 15 mg/mL D-luciferin was injected intraperitoneally and mice were analyzed with Photon Imager and M3 Vision Software for luciferase activity. ROIs were set on the entire tumors. Tumors were extracted 4 days after NIR-PIT for multiplex IHC and 5 days after NIR-PIT for flow cytometric analysis.

### 4.8. Multiplex Immunohistochemistry (IHC)

Extracted tumors were fixed with 10% formalin, embedded in paraffin and sliced in 4 μm thickness. The multiplex IHC was performed with an Opal 7-Color Automation IHC Kit (Akoya Biosciences, Hopkinton, MA, USA) and BOND RXm automated stainer (Leica Biosystems, Wetzlar, Germany) using the following antibodies: anti-pan-cytokeratin (CK) (rabbit poly; Bioss, Woburn, MA, USA), anti-CD8α (EPR20305; Abcam, Cambridge, United Kingdom), anti-CD44 (IM7; Bio X Cell, Lebanon, NH, USA), anti-F4/80 (D2S9R; Cell Signaling Technology, Danvers, MA, USA), anti-Ly6G (1A8; Bio X Cell, Lebanon, NH, USA), anti-CD11b (EPR1344; Abcam, Cambridge, United Kingdom) and anti-Gzmb (rabbit polyclonal; Abcam, Cambridge, United Kingdom). The slides were imaged in the Mantra Quantitative Pathology Workstation (Akoya Biosciences, Menlo Park, CA, USA). The multiplex IHC images were analyzed using inForm Tissue Finder software (Akoya Biosciences, Menlo Park, CA, USA). Cell phenotype was defined based on the antigen expressions as the following: CD8+ = CD8+ T cell, F4/80+ = macrophage, Ly6G+CD11b+ = neutrophil, Gzmb+CD3− = NK cell. Tissue phenotype was determined as the following: pan-cytokeratin positive = tumor. CD8+ T cells in the tumor area were counted as tumor-infiltrating CD8+ T cells.

### 4.9. Flow Cytometry

Single-cell suspensions of tumors were prepared for flow cytometry. Tumors were digested with collagenase type IV (1 mg/mL, Thermo Fisher Scientific, Waltham, MA, USA) and DNase I (20 μg/mL, Millipore Sigma, Burlington, MA, USA) and then gently dissociated and filtered with a 70 μm cell strainer (Corning, Glendale, AZ, USA). Cell suspensions were stimulated with Cell Activation Cocktail (with Brefeldin A) (BioLegend, San Diego, CA, USA), for 4 h. Then, the cells were stained with antibodies against CD3e (145-2C11; BioLegend, San Diego, CA, USA), CD8a (53-6.7; Thermo Fisher Scientific, Waltham, MA, USA) and LIVE/DEAD™ Fixable Far Red Dead Cell Stain Kit, for 633 or 635 nm excitation (Thermo Fisher Scientific, Waltham, MA, USA). Then, the cells were fixed and permeabilized with the Intracellular Fixation & Permeabilization Buffer Set (Thermo Fisher Scientific, Waltham, MA, USA) followed by staining with antibodies against Interferon-γ (IFN-γ) (XMG1.2; BioLegend, San Diego, CA, USA). The stained cells were evaluated with BD FACSCalibur and the data were analyzed with FlowJo software. Dead cells were gated out from analysis based on LIVE/DEAD dye staining.

### 4.10. In Vivo NIR-PIT with PD-1 Checkpoint Blockade

To evaluate the efficacy of the CD44-targeted NIR-PIT with anti-PD-1 antibody treatments, tumor-bearing mice were randomized into 4 groups as follows: (i) no treatment (Control); (ii) Injection of anti-PD-1 (PD-1); (iii) 100 μg of anti-CD44-IR700 i.v., NIR laser-light (690 nm, 150 mW/cm^2^, 50 J/cm^2^) exposure without anti-PD-1 (CD44-PIT); (iv) 100 μg of anti-CD44-IR700 i.v., NIR laser-light (690 nm, 150 mW/cm^2^, 50 J/cm^2^) exposure with anti-PD-1 (Combination). Injections of anti-CD44-IR700 were performed 6 days after tumor inoculation. NIR laser-light exposure was administered one day later. Anti-PD-1 (clone RMP1-14, Bio X Cell, 100–200 μg/injection as indicated in figures) was administered via intraperitoneal injection on days 6, 8, 10 and 12. Acute effects of the treatments were evaluated with BLI.

### 4.11. Re-Challenge with MOC2-Luc Cells

Mice whose tumors disappeared after combination CD44-targeted NIR-PIT and anti-PD-1 treatment were re-challenged via subcutaneous injection of 1 × 10^6^ MOC2-luc cells on the same side as the first tumor location 100 days after the first tumor injection. At the same time, mice with no previous injection were injected with same number of MOC2-luc cells in the caudal flank.

### 4.12. Statistical Analysis

Data are expressed as means ± SEM unless otherwise indicated. Statistical analysis was performed with GraphPad Prism (GraphPad Software, La Jolla, CA, USA). For a two-group comparison, an unpaired t-test was used. For multiple-group comparison, a one-way analysis of variance (ANOVA) followed by Tukey’s test was used. The cumulative probability of survival based on tumor volume was estimated with the Kaplan−Meier survival curve analysis and the results were compared with log-rank test followed by Bonferroni correction; *p*-values less than 0.05 were considered significant. 

## 5. Conclusions

NIR-PIT can convert a minimally immunogenic tumor into a highly immunogenic tumor by increasing the number and density of activated CD8+ T cells in tumor beds leading to improved efficacy with anti-PD-1 ICI. Thus, NIR-PIT can potentially be used as a neoadjuvant enhancer of immunogenicity for immune checkpoint inhibition. Moreover, this study hints at the possibility that the combination of NIR-PIT and anti-PD-1 ICI can lead to establishment of long-term immune memory to prevent future recurrences.

## Supplementary Materials

The following are available online at https://www.mdpi.com/2072-6694/12/12/3747/s1, Figure S1: Cell number of F4/80+ cells within three tumors was counted in multiplex IHC images, Figure S2: CD44 expression of CD8+ T cells within MOC2-luc tumor, Figure S3: Cell number of F4/80+, Ly6G+11b+ and Gzmb+CD3− cells within tumor tissue before and after CD44-targeted NIR-PIT for MOC2-luc tumors were counted in multiplex IHC images.
