# Supplementary material for: Increased Immunogenicity of a Minimally Immunogenic Tumor after Cancer-Targeting Near Infrared Photoimmunotherapy
Source: Cancers (Basel). 2020 Dec 12;12(12):3747. doi: 10.3390/cancers12123747 (PMC7763141; doi:10.3390/cancers12123747)
Supplement: Supplementary file 1 [file cancers-12-03747-s001.pdf]

Supplementary Materials: Increased Immunogenicity of a Minimally Immunogenic Tumor after Cancer-Targeting Near Infrared Photoimmunotherapy

Hiroaki Wakiyama, Aki Furusawa, Ryuhei Okada, Fuyuki Inagaki, Takuya Kato, Yasuhiro Maruoka, Peter L. Choyke and Hisataka Kobayashi

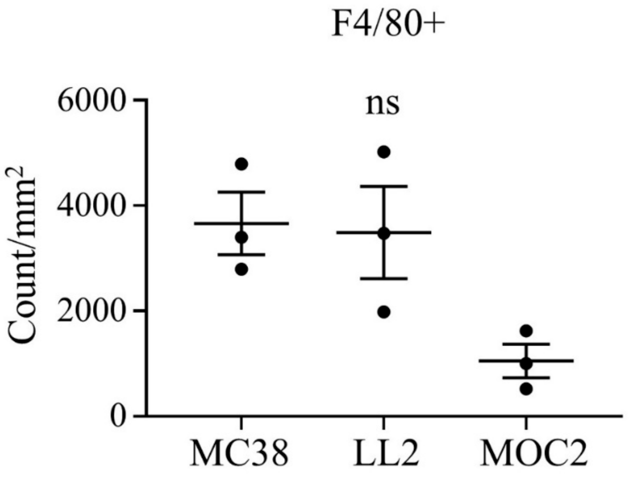

Figure S1. Cell number of F4/80+ cells within three tumors was counted in multiplex immunohistochemistry images. Data were shown as cell count per mm² (n = 3; ns, not significant; one-way ANOVA followed by Tukey's test).

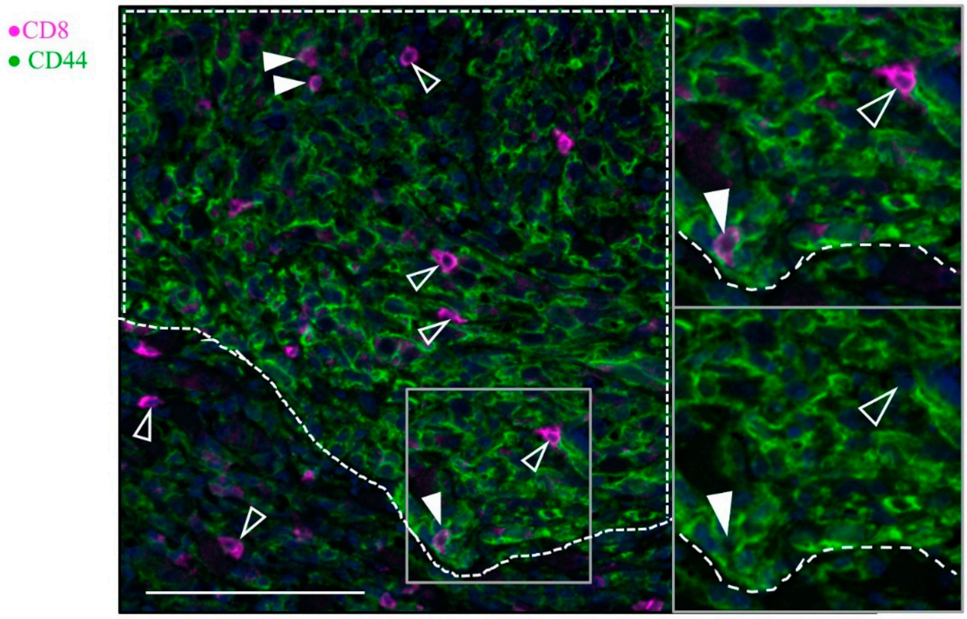

Figure S2. CD44 expression of CD8+ T cells within MOC2-luc tumor. A representative multiplex immunohistochemistry image of MOC2-luc tumor. CD8 expression and CD44 expressions are shown

in magenta and green respectively. The tumor area is enclosed with dotted white line. Right upper inset shows an enlarged composite image of the area enclosed in rectangle. Right lower inset shows an enlarged single channel image of CD44 staining of the area enclosed in rectangle. Examples of CD8+/CD44+ cell and CD8+/CD44− are indicated with white filled arrowheads and open arrowheads respectively. Scale bar = 100 μm.

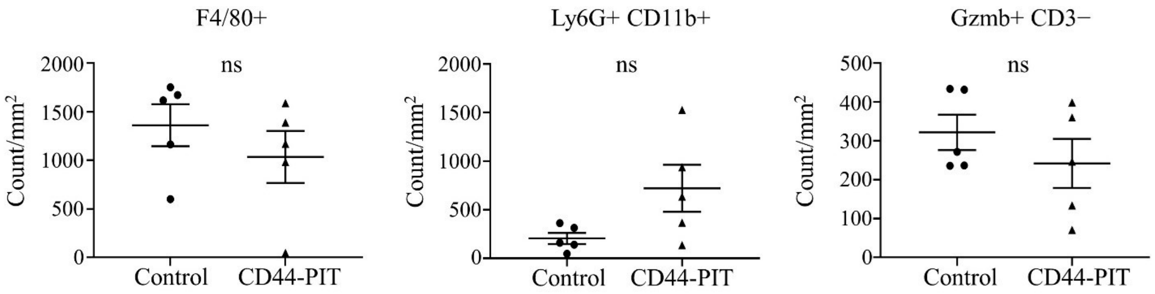

Figure S3. Cell number of F4/80+, Ly6G+/CD11b+, and Gzmb+/CD3− cells within tumor tissue before and 4 days after CD44-targeted NIR-PIT for MOC2-luc tumors were counted in multiplex immunohistochemistry images. Data were shown as cell count per mm^2 ($n = 5$; ns, not significant; one-way ANOVA followed by Tukey's test). Control, no treatment; CD44-PIT, i.v. injection of anti-CD44-IR700 with NIR light exposure. Each dot represents independent experiments. Vertical lines and error bars represent mean and SEM.

© 2020 by the authors. Licensee MDPI, Basel, Switzerland. This article is an open access article distributed under the terms and conditions of the Creative Commons Attribution (CC BY) license (http://creativecommons.org/licenses/by/4.0/).
